# Genome-wide endogenous DAF-16/FOXO recruitment dynamics during lowered insulin signalling in *C. elegans*

**DOI:** 10.18632/oncotarget.6282

**Published:** 2015-11-02

**Authors:** Neeraj Kumar, Vaibhav Jain, Anupama Singh, Urmila Jagtap, Sonia Verma, Arnab Mukhopadhyay

**Affiliations:** ^1^ Molecular Aging Laboratory, National Institute of Immunology, Aruna Asaf Ali Marg, New Delhi, India; ^2^ Current address: Centre for Human Genetics and Molecular Medicine, School of Health Sciences, Central University of Punjab, Bathinda, India

**Keywords:** DAF-16, FOXO, ChIP-seq, C. elegans, transcription, Gerotarget

## Abstract

Lowering insulin-IGF-1-like signalling (IIS) activates FOXO transcription factors (TF) to extend life span across species. To study the dynamics of FOXO chromatin occupancy under this condition in *C. elegans*, we report the first recruitment profile of endogenous DAF-16 and show that the response is conserved. DAF-16 predominantly acts as a transcriptional activator and binding within the 0.5 kb promoter-proximal region results in maximum induction of downstream targets that code for proteins involved in detoxification and longevity. Interestingly, genes that are activated under low IIS already have higher DAF-16 recruited to their promoters in WT. DAF-16 binds to variants of the FOXO consensus sequence in the promoter proximal regions of genes that are exclusively targeted during low IIS. We also define a set of ‘core’ direct targets, after comparing multiple studies, which tend to co-express and contribute robustly towards IIS-associated phenotypes. Additionally, we show that nuclear hormone receptor DAF-12 as well as zinc-finger TF EOR-1 may bind DNA in close proximity to DAF-16 and distinct TF classes that are direct targets of DAF-16 may be instrumental in regulating its indirect targets. Together, our study provides fundamental insights into the transcriptional biology of FOXO/DAF-16 and gene regulation downstream of the IIS pathway.

## INTRODUCTION

The evolutionarily conserved IIS pathway controls metabolism, development, stress response and longevity across the animal kingdom [1]. The Forkhead TFs (FOXO) play a critical role in sculpting the transcriptional topology downstream of the IIS in worms, flies and mammals. Considering the conservation of IIS, simple model organisms like *Caenorhabditis elegans* are instrumental in finding how FOXO recruits to its direct transcriptional targets to regulate gene expression in a context-dependent manner.

In *C. elegans*, mutations in the IIS receptor *daf-2* lower IIS leading to a dramatic increase in life span, stress tolerance, higher fat stores and tendency to arrest at an alternate developmental stage called dauer [1“^4^]. All these phenotypes are dependent on the single FOXO homolog, DAF-16. Only two studies have previously reported the genome-scale recruitment profile of DAF-16/FOXO under conditions of low IIS using DamID or ChIP-sequencing (ChIP-Seq) [5, 6]. However, both these studies used transgenic worms overexpressing a single isoform of DAF-16 tagged to GFP that may not precisely reproduce the endogenous situation, making it difficult to evaluate the role of the TF. Although these studies have provided indications towards the complexity of gene regulation by DAF-16, more detailed analysis is required to elucidate how this transcription factor works in the endogenous settings. In our previous study, we used an anti-DAF-16 antibody to immunoprecipitate chromatin-bound DAF-16/FOXO and identified 33 direct target genes [7]. But the study failed to saturate the genome due to its low throughput nature. In this context, a genome-wide recruitment study in a non-manipulated worm will help tremendously in understanding *in vivo* DAF-16/FOXO transcriptional biology.

Here we report the first global chromatin recruitment dynamics of endogenous DAF-16/FOXO under wild-type (WT) and low IIS conditions using ChIP-Seq. Our data shows significantly more enrichment of DAF-16 binding compared to previous ChIP-seq using an overexpression strain [6] and we report ∼4000 new binding events. We also present a more detailed analysis of the recruitment profile compared to previous studies. Interestingly, we find that genes that are activated under low IIS condition already have higher DAF-16 recruitment on their promoters in WT. Surprisingly, these genes are transcribed at a higher level compared to genes to whose promoters DAF-16 recruit only during low IIS. Comparing our data to other studies, we define a ‘core’ set of DAF-16 direct targets that we validate phenotypically for their contributions towards IIS pathway-dependent phenotypes; these targets will serve as an important resource for future studies on DAF-16/FOXO. Importantly, we show that DAF-16, *Drosophila* dFOXO and human FOXO3 bind orthologous genes when activated. Using this data, we identify TFs that may bind in close proximity of DAF-16 during lowered IIS conditions. Finally, we identify specific classes of TFs directly regulated by DAF-16 that may modulate the expression of DAF-16 indirect targets. Together, our analysis provides a robust framework to study the endogenous transcriptional dynamics of DAF-16/FOXO and provides a glimpse into the complexity of gene regulation downstream of the IIS pathway.

## RESULTS AND DISCUSSION

### Endogenous DAF-16/FOXO recruitment dynamics during low IIS

**Figure 1:**
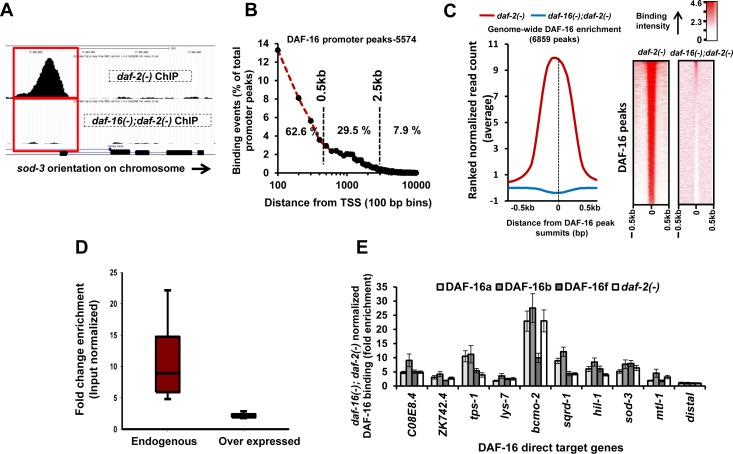
Genome-wide recruitment profile of DAF-16/FOXO **A.** A DAF-16 peak on *sod-3* promoter in *daf-2(−)* is absent in *daf-16(−);daf-2(−).* **B.** Distribution of DAF-16 peaks with respect to TSS. **C.** Enrichment of ranked normalized reads at the DAF-16 peak summits (left panel) and its heat map representation (right panel) in *daf-2(−)* that is absent in *daf-16(−);daf-2(−).* **D.** DAF-16 enrichment in *daf-2(−)* normalized to input samples. Enrichment was calculated using MACS in DAF-16 peaks that were common between this study (Endogenous DAF-16) and that of Riedel et al. (2013) (Overexpressed DAF-16). **E.** Recruitment profiles of DAF-16 isoforms in *daf-2(−)* compared to *daf-16(−);daf-2(−)* as determined by ChIP-Q PCR. DAF-16a, DAF-16b or DAF-16f represents transgenic lines where only one of the DAF-16 isoforms is expressed in a *daf-16(−);daf-2(−)* background [9].

To uncover the chromatin occupancy pattern of endogenous DAF-16/FOXO, we generated a ChIP-grade antibody against the soluble protein. ChIP-qPCR using primers designed to amplify the promoter proximal region of *sod-3*, a bonafide DAF-16/FOXO direct target [7], showed that DAF-16-bound chromatin is enriched in the immune-complex immunoprecipitated from *daf-2(−)* as compared to the one from *daf-16(−);daf-2(−)* (Figure S1A). Such robust enrichment was not observed in a distal region of the gene. Validated ChIP-ed DNA were used as templates to prepare single-end ChIP-sequencing libraries (Illumina Inc., USA) that also retained the enrichment on *sod-3* promoter as above (Figure S1A). Following deep sequencing, we obtained 6860 input-normalized peaks (*P* < 1×10^−5^, FDR < 5%) in case of *daf-2(−)* as against one significant peak in *daf-16(−);daf-2(−)*, showing the specificity of the ChIP experiments (Table S1; also Figure S2). As expected, we observed a single DAF-16 peak in the promoter of *sod-3* while no peak was detected in the 3′ region or in *daf-16(−);daf-2(−)* (Figure 1A).

Majority of the DAF-16 peaks (5574) were positioned within the 0.5 kb region upstream of the transcription start site (TSS) (Figure 1B, S1B). About 68.4% or 4696 peaks in *daf-2(−)* were assigned to 3734 coding genes while the remaining were in the vicinity of non-coding genes, indicating extensive regulatory role of the TF (Table S2). This is also reflected in the distribution of DAF-16 peaks on the chromosomes that show enrichment on non-coding genes in Chr I, II, III, IV and X (Figure S1C). The mean read density (MRD) distribution analysis around the DAF-16 peak summits (±0.5 kb) shows clear enrichment within a narrow window of ∼200 bp in *daf-2(−)* that is absent in *daf-16(−);daf-2(−)* (Figure 1C). Together, using a robust ChIP-seq procedure, we have generated the first endogenous genome-wide DAF-16/FOXO recruitment profile under low IIS conditions.

Previous studies to chart genome-wide DAF-16 binding used overexpression strains [5, 6]. We compared our data with these studies and report a large number of new genes with DAF-16 binding peaks in the promoter proximal region (4389 genes) (Figure S1D). This was surprising as we expected that DAF-16 overexpression would lead to more bound targets compared to endogenous DAF-16. This unexpected observation may be explained partly by the fact that we achieved more enrichment on the DAF-16 binding loci compared to the previous ChIP-seq study [6] (Figure 1D, S1E). The previous study used overexpression of only the DAF-16a isoform [8] for the ChIP-seq experiments. This may result in lower enrichment compared to our study since the antibody detects all the DAF-16 isoforms and reports the DAF-16 binding dynamics more accurately. Additionally, we used a mixed stage worm culture to capture maximum binding events while Riedel *et al.* used L4-staged worms. Together, endogenous ChIP-seq reported in this study may provide a more realistic estimate of DAF-16 recruitment profile under low IIS condition.

The *daf-16* gene codes for several isoforms; three of them are well-characterized and have overlapping as well as distinct functions [9]. To determine the relative recruitment dynamics of the DAF-16 isoforms, we used transgenic worms where only one isoform of DAF-16, i.e., DAF-16a, DAF-16b or DAF-16f has been rescued in *daf-16(−);daf-2(−)* [9]. ChIP-PCR analysis revealed that all DAF-16 isoforms bind DNA. However, the DAF-16b had comparatively higher binding to all the promoters, although it plays only a modest role in pharynx remodelling [10](Figure 1E). Thus, DAF-16b may have other undiscovered functions or alternatively, the dynamics of chromatin recruitment may change in scenarios where only one isoform is present.

### TSS proximity of binding defines the strength of DAF-16/FOXO transcriptional response

**Figure 2:**
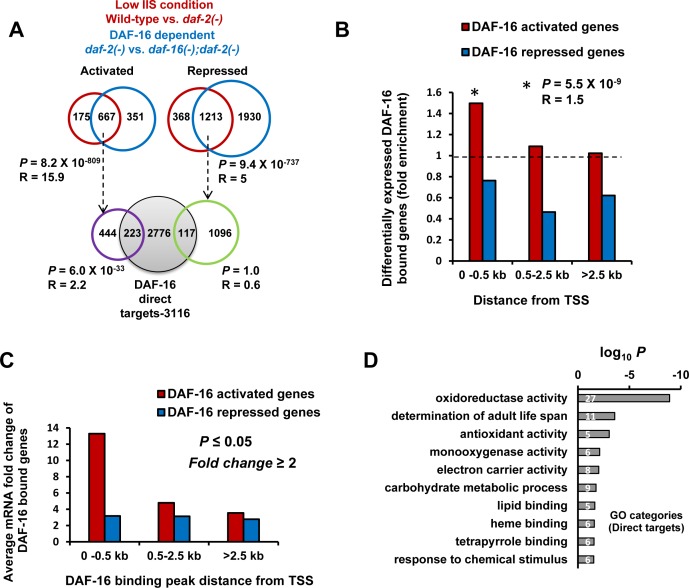
Promoter-proximal binding of DAF-16 ensures optimal transcriptional response **A.** Genes activated (N=667) or repressed (N=1213) in *daf-2(−)* in a DAF-16-dependent manner were overlapped with genes in which DAF-16 binds within 2.5 kb promoter region upstream of TSS (Grey circle; lower panel) to obtain 223 significant (Hypergeometric test) DAF-16 direct targets that are transcriptionally relevant. **B.** DAF-16-dependent genes that have peaks located within 0.5 kb of TSS are more likely (Hypergeometric test) to be activated in *daf-2(−)*. **C.** Average mRNA fold change of genes activated or repressed in *daf-2(−)*. The genes were categorized based on distance of DAF-16 peaks from TSS. **D.** Gene annotation enrichment analysis highlights biological functions of genes directly activated by DAF-16.

To correlate DAF-16 chromatin recruitment to its transcriptional efficiency, we first compared expression profiles of WT, *daf-2(−)* and *daf-16(−);daf-2(−)* strains by RNA-seq (Table S3). This identified 667 genes that were up- (activated) and 1213 genes down-regulated (repressed) during low IIS compared to WT, in a *daf-16*- dependent manner (fold change ≥2; *P*≤0.05) (Figure 2A, upper circles). Among the activated genes, only 223 (*R* = 2.2, *P* = 6.0×10^−33^) are direct targets of the TF (Figure 2A, lower circles). However, no significant overlap with binding data was observed in case of repressed genes, supporting previous suggestions that DAF-16 may act primarily as an activator [5, 6, 11]. The majority of the genes that are activated in *daf-2(−)* are indirect targets of DAF-16 as they lacked a DAF-16-binding peak in the promoter proximal region, suggesting a hierarchical control involving multiple downstream TFs. It appears that DAF-16 may be “parked” at multiple locations on the chromatin without apparent transcriptional activity, similar to dFOXO [12]. It is tempting to speculate that some of these binding may represent enhancer sites that regulate expression of distant genes. It is also possible that these genes may be transcribed at a low rate or in a tissue or context-specific manner.

We found that genes with binding peaks within 0.5 kb of TSS are more likely to be activated in *daf-2(−)* (*R* = 1.5; *P* = 5.5×10^−9^) (Figure 2B) with the highest average mRNA fold change (Figure 2C). However, this was not the case with genes that are repressed (Figure 2C). Moreover, a single peak of DAF-16 in the promoter-proximal region resulted in higher transcriptional induction compared to two or more peaks (Figure S1F). Together, our data suggests that DAF-16/FOXO acts predominantly as an activator and TSS proximity correlates directly to transcriptional efficiency.

### DAF-16 direct targets are enriched for genes involved in detoxification and longevity

We find that DAF-16 directly binds and transcriptionally regulates only a small subset of the genes that are upregulated during low IIS. These genes may comprise the immediate early response to lowering of IIS; the indirect targets possibly represent the outcome of a hierarchical response that may be relatively delayed in onset. So, to understand the biological role of the DAF-16/FOXO direct targets, we used DAVID [13] for Gene Ontology analysis. The direct and transcriptionally relevant activated targets (223) were found to be enriched in genes having oxidoreductase, antioxidant and monooxygenase activity with role in determination of adult life span (Figure 2D). On the other hand, DAF-16 indirect targets are also enriched in genes involved in metabolic processes (Figure S3). It is possible that worms respond to lowering of IIS by first upregulating the detoxification machinery through DAF-16 that is primarily responsible for life span extension. The secondary genes that are upregulated indirectly may be required to support the enhanced longevity.

### DAF-12 and EOR-1 may bind chromatin at close proximity to DAF-16 during low IIS

**Figure 3:**
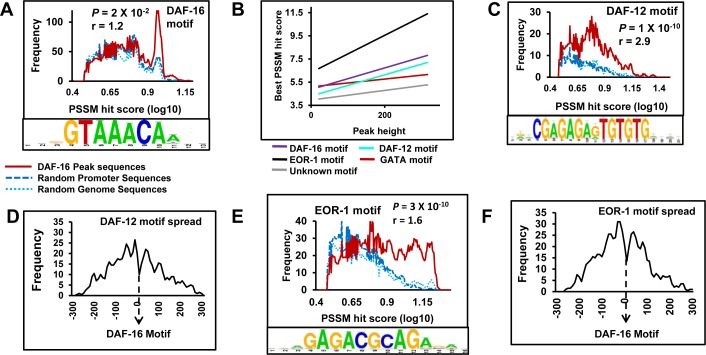
DAF-12 and EOR-1 may bind chromatin at close proximity to DAF-16 during low IIS **A.** Upper panel shows the frequency of DAF-16 motif (red) within the DAF-16 peaks as compared to random sequences (blue). Lower panel contains the consensus DAF-16 motif identified by RSAT. **B.** Correlation of best PSSM hit scores of DAF-16, EOR-1, DAF-12, GATA or an unknown motif with DAF-16 peak heights. **C.** Upper panel shows the frequency of DAF-12 motif (red) within the DAF-16 peaks as compared to random sequences (blue). Lower panel contains the consensus DAF-12 motif identified by RSAT. **D.** Distribution of DAF-12 motifs with respect to DAF-16 motifs in *daf-2(−).* **E.** Upper panel shows the frequency of EOR-1 motif (red) within the DAF-16 peaks as compared to random sequences (blue). Lower panel contains the consensus EOR-1 motif identified by RSAT. **F.** Distribution of EOR-1 motifs with respect to DAF-16 motifs in *daf-2(−). P* values calculated using unpaired student's *t* test.

As discussed above, lowering IIS leads to enhanced longevity, elevated stress tolerance and increased dauer formation. Although all these phenotypes are dependent on a functional DAF-16, other transcriptional regulators are also known to have important role to play in determining these phenotypes. For e.g., HSF-1 and SMK-1 are known to influence *daf-2(−)* longevity and/or stress tolerance [14, 15]. On the other hand, DAF-12 is required for enhanced dauer formation as well as longevity [16, 17]. In order to identify transcriptional regulators that may co-regulate DAF-16 direct targets, we searched for signatures of DNA-binding factors within the DAF-16 peaks. *De novo* motif search [18] revealed, as expected, the enrichment of 5′-sygGTAAACAasr-3′ motif in 71.2% of the DAF-16 peaks (Figure 3A). This motif had strong Pearson correlation (0.998) with DAF-16 reference motif 5′-GTAAACA(A)-3′ [11, 19, 20] (Figure 3A, lower panel). We used the position-specific scoring matrices (PSSMs) of this motif and scanned all DAF-16 peak regions (peak summits ± 250 bp) in our data to show that the matching frequency of DAF-16 motif is significantly higher as compared to the random sequences, specifically in the region of the higher PSSM hit score (Figure 3A, upper panel, S5A), indicating a robust enrichment of DAF-16 motifs. Importantly, the DAF-16 motifs were found to be distributed around the DAF-16-binding peaks (Figure S4A).

This analysis also revealed the presence of GATA-like motifs (present in 61.1 % of the DAF-16 peaks), a prospective DAF-12-binding motif (present in 40.4% of the peaks) as well as an EOR-1-binding motif (present in ∼50% peaks), apart from unknown low-complexity motifs (Figure 3C, 3E, S5B, C, lower panels). However, the GATA motif and an unknown low complexity motif did not show much enrichment as compared to the random sequences (Figure S4B, C, upper panels). On the other hand, DAF-12 as well as the EOR-1 motif was overrepresented in the DAF-16 peaks across the range of PSSM hit score (Figure 3C, 3E, upper panel, S5B, C). Additionally, best PSSM hit scores for DAF-16, EOR-1 as well as DAF-12 motifs correlated better with DAF-16 peak height, compared to the GATA or the unknown motif (Figure 3B), suggesting that the occurrence of DAF-16, EOR-1 or DAF-12 motifs may ensure higher recruitment of DAF-16. Further, the DAF-12 as well as EOR-1 motifs within the DAF-16 peaks are tightly centred around the DAF-16 motifs (Figure 3D, 3F). Together, EOR-1 and DAF-12 can potentially bind chromatin at close proximity to DAF-16 under conditions of low IIS to regulate gene expression.

DAF-12 is a nuclear receptor that is homologous to the vertebrate farnesoid-X (FXR), liver-X and vitamin-D receptors. It binds to bile acid-like steroids known as the dafachronic acids (DAs), which regulate its transcriptional activity [21, 22]. DAF-12 acts as a molecular switch downstream of the IIS pathway (DAF-16/FOXO) and TGF-beta like pathway (DAF-3/SMAD and DAF-5/SKI) to determine the choice between dauer formation and reproductive growth [23]. DAF-12 affects multiple DAF-16-dependent phenotypes like dauer and longevity. Although these two factors are known to interact in worms and mammals, the molecular mechanism is less clear [24]. The *eor-1* encodes the ortholog of human PLZF, a BTB/zinc-finger transcription factor and functions downstream of the EGF pathway to regulate longevity [25]. The EGF and IIS pathways work in parallel, responding to different physiological or environmental cues to maintain protein homeostasis [26]. Our finding that DAF-12 and EOR-1 also may bind DAF-16 direct target genes suggests a novel mechanism by which hormone signalling and IIS pathway can impinge on the promoters of direct targets to co-ordinately regulate gene expression. This needs to be verified at the transcriptional and physiological level in future.

### DAF-16 binds to variants of FOXO consensus sequence under low IIS

**Figure 4:**
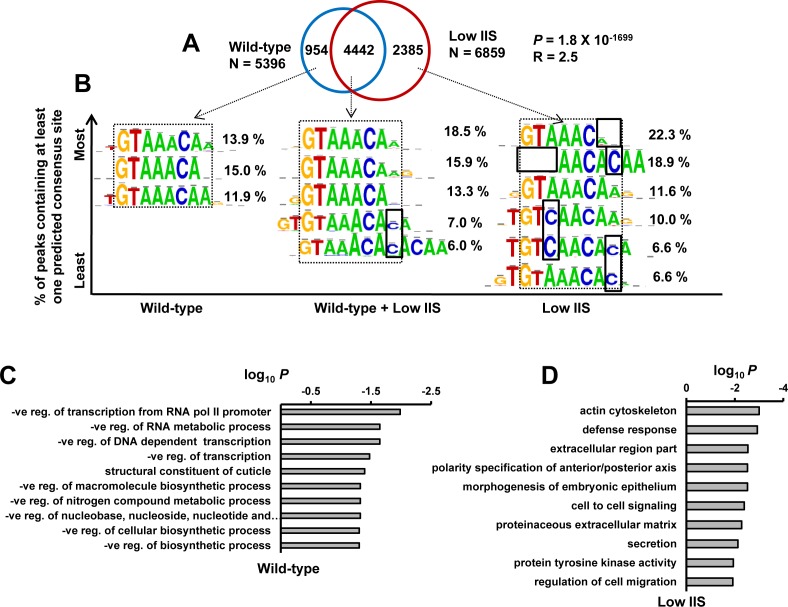
DAF-16 binds to variants of core FOXO consensus sequence on genes that it recruits to exclusively under low IIS **A.** Overlap of DAF-16 binding peaks in WT and *daf-2(−)*. Many new peaks (2385) appear in *daf-2(−)*. *P* calculated using Hypergeometric test. **B.** FOXO consensus sequence was identified by RSAT *de novo* motif finding tool in DAF-16 binding peaks (within ± 250 bp of summit) for genes that the transcription factor recruits to in WT, *daf-2(−)* or both. Percentage occurrence of each consensus sequence is shown. **C.**-**D.** Gene annotation enrichment analysis highlights biological functions of genes that DAF-16 recruits to exclusively in WT **C.** or *daf-2(−)* **D.**

FOXO/DAF-16 is known to be majorly regulated at the level of nuclear translocation through phosphorylation by its upstream kinases [27]. To determine whether nuclear-cytoplasmic distribution of DAF-16 is reflected in its recruitment pattern, we compared the binding dynamics of *daf-2(−)* with WT (Figure 4A). We find that DAF-16 binds to exclusive targets in WT (954) and *daf-2(−)* (2385) as well as a large number of common targets (4442). The relative positions of the common DAF-16 binding peaks in the two strains do not shift and exhibit normal distribution with respect to TSS (Figure S6A). This is in contrast to the observation in *Drosophila* [12], where only the extent of dFOXO binding changes at the target loci in WT vs a low IIS mutant. Together, the DAF-16 recruitment dynamics partially reflect the nuclear-cytoplasmic distribution as not all DAF-16 may be excluded from the nucleus under WT condition. Since DAF-16 has multiple isoforms, it is also possible that one of the isoforms may be mostly chromatin bound while others shuttle between nucleus and cytoplasm in a context-dependent manner. Since our antibody recognizes all the isoforms, we were not in a position to resolve this mode of regulation. In line with this idea, the DAF-16f isoform is evenly distributed in the cytosol and nucleus even under the low IIS condition [9].

Next, we focused on the FOXO consensus sequences in the DAF-16 peaks found exclusively in WT or *daf-2(−)* as well as from those commonly observed in the two strains. DAF-16 peaks, present exclusively in WT, were near match to the core FOXO consensus sequence [11, 28] (Figure 4B, left). These genes were found to function mainly in negative regulation of transcription and metabolic processes, as determined by Gene Ontology analysis using DAVID [13] (Figure 4C). Interestingly, in DAF-16 peaks exclusive to *daf-2(−)*, several variants of the FOXO consensus sequences were found apart from the core consensus motif (Figure 4B, right). These categories of genes functions mostly in defence response, cell to cell signalling etc (Figure 4D). This suggests that activated DAF-16, as in *daf-2(−)*, may have higher binding affinity and bind imperfect FOXO consensus sequences. Alternatively, they may require other factors to assist in binding.

### Genes activated in *daf-2(−)* already have higher DAF-16 recruitment in WT

**Figure 5:**
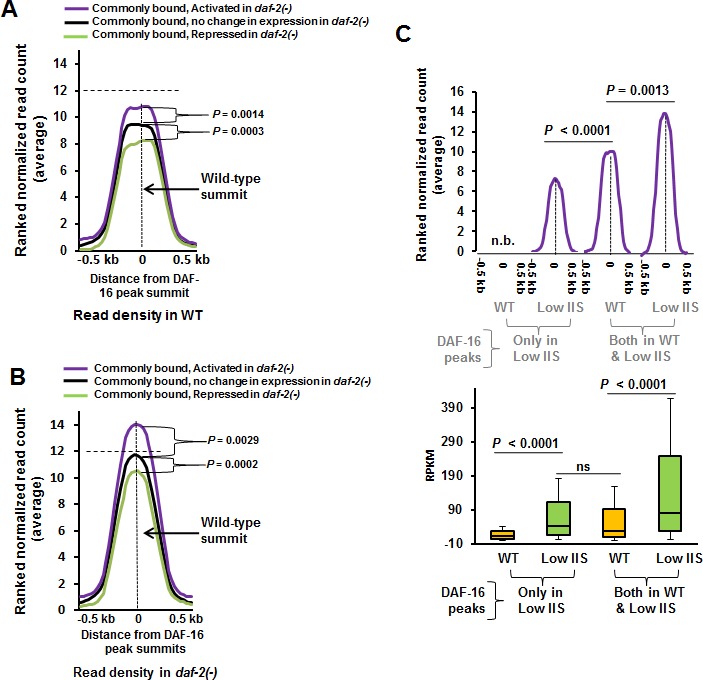
Genes that are activated under low IIS have higher DAF-16 recruitment in WT **A.** Genes that are activated in *daf-2(−)* have higher DAF-16 recruitment on their promoters in WT compared to genes whose expression remain unchanged or are repressed. Ranked normalized read counts in WT were plotted against the distance from the peak summits (± 0.5 kb). Coding genes that are DAF-16-bound both in WT and *daf-2(−)* (2506) were considered. They were categorised as activated, repressed or no change based on their expression in *daf-2(−)* compared to WT. *P* calculated using Mann Whitney test. **B.** Ranked normalized read counts in *daf-2(−)* were plotted against the distance from the peak summits (± 0.5 kb) similar to **A.** *P* calculated using Mann Whitney test. **C.** RPKM of genes that are activated under low IIS condition as in *daf-2(−)* compared to WT (lower panel). Genes are categorized based on the fact that DAF-16 binds to the promoters exclusively in *daf-2(−)* or commonly in both WT as well as in *daf-2(−)*. *P* between WT and low IIS calculated by Wilcoxon signed rank test. *P* between low IIS (DAF-16 peaks only in Low IIS) and WT (DAF-16 peaks both WT and Low IIS) in calculated by Mann Whitney test. The corresponding ranked normalized read counts are provided in the upper panel. *P* calculated using Mann Whitney test. n.b. indicates no binding peaks observed.

To further study the dynamics of DAF-16 recruitment in normal and low IIS conditions, we correlated the extent of binding of the transcription factor to chromatin in WT and *daf-2(−)* with gene activation levels. We observed that genes that are activated in *daf-2(−)* already have higher DAF-16 recruitment in WT worms compared to genes whose expression remain unchanged, while those that are repressed have lower (Figure 5A). In *daf-2(−)*, the average peak heights of all the three categories increase, but only a subset is transcriptionally upregulated with respect to WT (Figure 5B). Thus, it appears that the genes that are destined to be activated under low IIS are already marked by presence of more DAF-16 on their promoter proximal regions (Figure 5B). More interestingly, no such difference in binding was noticed for genes that are bound by the transcription factor exclusively in *daf-2(−)* (Figure S6B). These genes may require additional factors to modulate gene expression.

Above, we observed two different dynamics of DAF-16-dependent gene activation under low IIS; one where DAF-16 binds to promoters in both WT as well as in *daf-2(−)*, the other where DAF-16 binds promoters exclusively in *daf-2(−)*. We asked whether these two scenarios lead to different levels of gene expression. We compared the RPKM of the genes under these two categories as determined by transcriptomics and only observed clear positive correlation between binding and activation of gene expression; no correlation was found in case of genes that are repressed or unchanged (Figure S6C). We find that in case of genes where DAF-16 binds in both WT and *daf-2(−)*, there is more robust gene transcription as compared to ones where DAF-16 binds exclusively under low IIS condition, as in *daf-2(−)* (Figure 5C, lower panel). This may not be directly attributed to the levels of recruitment as increased DAF-16 binding does not necessarily translate into more transcription [compare Figure 5C, blue with orange]. Importantly, this does not significantly affect the fold change in gene expression (data not shown). Put together, genes that are commonly bound by DAF-16 in WT and *daf-2(−)* are transcribed at a higher level in *daf-2(−)* compared to the ones to which the transcription factor recruits to under low IIS.

### ‘Core’ DAF-16 direct targets are often co-regulated and contribute robustly towards IIS pathway-dependent phenotypes

**Figure 6:**
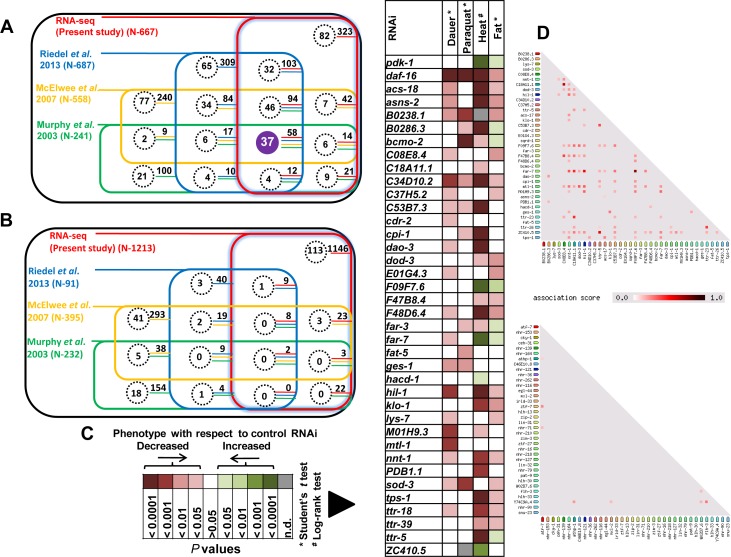
DAF-16 “core” direct targets contribute robustly towards IIS-regulated phenotypes **A.** Comparison of DAF-16 ChIP-seq with transcriptomics data from multiple studies [6, 19, 29] reveals 37 “core” direct DAF-16 targets that are activated in *daf-2(−)*. Each coloured square represents a RNA-seq or microarray data taken from the indicated studies. Genes that were found to be common with our ChIP-seq data are highlighted by dotted circles (direct DAF-16 targets). The numbers adjoining the dotted circles represent genes that overlap with our transcriptomics data. **B.** No significant overlap was observed in case of repressed genes. **C.** IIS pathway-dependent phenotypes are differentially affected when the “core” DAF-16 targets are knocked down by RNAi. The *P* values (obtained either by Student's *t* test or log rank test) of significantly affected genes are plotted. Details of the phenotypic analysis experiments provided in Table S7. **D.** DAF-16 core direct target genes are co-expressed with each other as determined by STRING database analysis (upper panel). No such co-expression was observed in case of a randomly chosen set of 37 genes.

DAF-16 is a central regulator of gene expression and is involved in multiple biological processes [4]. Studies involving this TF often require experiments that follow its direct target genes transcriptionally as well as phenotypically. So using our dataset, we defined a ‘core’ set of DAF-16/FOXO direct and transcriptionally relevant targets by comparing our study to several previous studies that employed transcriptomics or microarray [6, 19, 29] (Figure 6A, 6B). We found 37 activated genes that overlap with all these previous studies and represent the “core” direct targets that are highly relevant (Figure 6A). No such significant overlap was observed among DAF-16 repressed genes (Figure 6B).

The *daf-2(−)* worms have increased stress tolerance, enhanced longevity, higher fat storage and propensity to arrest as dauers [30]. All these phenotypes are completely dependent on DAF-16. To evaluate the contributions of DAF-16 direct targets on these phenotypes, we systemically knocked down each one of them by RNAi in *daf-2(−)*. We found that most of the genes affect multiple phenotypes in *daf-2(−)* (Figure 6C), indicating that DAF-16 direct targets play important roles during low IIS. Importantly, these genes will now serve as essential reagents for pursuing *bonafide* DAF-16 direct targets for analysis.

Genes that function together often co-express [31]. We analyzed the co-expression profile of the ‘core’ DAF-16 direct targets using STRING database Version 10 (www.string-db.org/) (Figure 6D). We found that several of these genes are co-expressed under multiple conditions and may suggest linked functions. The *nnt-1* (putative proton-pumping nicotinamide nucleotide transhydrogenase), *far-7* (fatty acid/retinol binding protein) and *hil-1* (histone H1-like protein) are some of the annotated genes that show co-expression with other genes. This type of association may not occur by chance as in 5 sets of randomly generated list of 37 genes, co-expression was not observed (Figure 6D, S7). Thus, DAF-16 preferentially targets genes that are co-expressed to modulate phenotypes downstream of the IIS pathway.

### Conservation of FOXO direct targets in worms, flies and human

**Figure 7:**
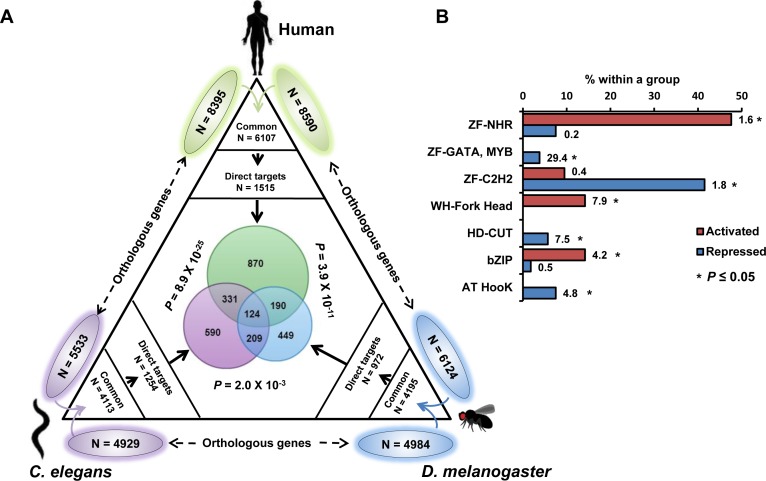
FOXO recruitment to its target genes is conserved Orthologous genes between a pair of species is shown outside the triangle. Common orthologous genes were overlapped with the binding data (direct targets) from either human FOXO, dFOXO or DAF-16 (this study) as indicated. Species-specific direct targets were overlapped and are shown in the centre of the triangle along with *P* values calculated using Hypergeometric test. **B.** Relative enrichment of different types of transcription factors that are directly activated or repressed by DAF-16. Numbers indicate R = representation score. *P ≤ 0.05 by Hypergeometric test.

The FOXO TF works in the adipocytes/fat bodies/intestinal cells to modulate life span in mice, flies and worms [32“^34^]. To determine whether the direct targets of DAF-16 are conserved, we compared our data with that of human FOXO3 and fly dFOXO binding data-sets [35] [12] (Figure 7A). Among the orthologous genes between *C. elegans, Drosophila* and humans, 124 were found to be common and represent genes that are bound by FOXO in these species under low IIS conditions. Interestingly, these genes are enriched for GO terms involving vesicle-mediated endocytosis/membrane organization (Enrichment Score or ES 3.18), regulation of Rab protein signalling (ES 2.91), reproduction (ES 2.18), cell motility (ES 2.14), motor proteins (ES2.03), neurogenesis (ES 1.77), regulation of organismal growth (ES 1.41) etc. (Dataset 1). These genes may play important role in mediating the prolongevity effects associated with lowered IIS. Together, FOXO TFs bind an overlapping set of genes in worms, flies and human.

### Transcription factors downstream of DAF-16 that it directly regulates

Comparing DAF-16 binding data to transcriptomics analysis indicates that DAF-16 directly regulates only a small fraction of the *daf-2(−)* transcriptome. Therefore, TFs within the directly targeted genes may act as second tier regulators that control the indirect targets of DAF-16. We found 21 TFs among upregulated and 53 TFs among down-regulated direct target genes (Figure 7B, Tables S4, S5). Interestingly, certain categories of transcription factors are enriched in each case. For example, among the activated genes, the winged helix forkhead TFs, the b-ZIP TFs and the zinc-finger nuclear hormone receptors are enriched. On the other hand, zinc finger containing GATA factors, C2H2 zinc-finger TFs, homeodomain CUT-like TFs and AT Hook TFs are enriched in the downregulated direct targets. Thus, DAF-16 may employ distinct categories of downstream TFs to coordinately control the indirect targets.

## CONCLUSION

Based on this study, it appears that DAF-16 in general or some of its isoforms may remain parked in the immediate promoter proximal regions of genes that it regulates; the ones that it will eventually activate are marked with more bound transcription factor. During low IIS, due to influx of more DAF-16 molecules into the nucleus, binding at these sites increase while new regions are also recruited to. Interestingly, there is a differential requirement for FOXO consensus sequence for binding of DAF-16, with the regions that are exclusively recruited to in *daf-2(−)* having considerable variation. It is possible that different isoforms of DAF-16 have dissimilar binding consensus and may be assisted by other factors to promote/oppose binding. Our study thus highlights the complexity of gene regulation downstream of the IIS that is controlled by DAF-16/FOXO. With multiple isoforms that localize distinctly and modulate gene expression differentially, DAF-16 recruitment studied using a single isoform will not be sufficient to reveal the detailed mechanism of gene regulation by the transcription factor. On the other hand, our study using an anti-DAF-16 antibody that recognizes all the isoform will not have the adequate resolution to dissect isoform-specific regulation. In future, we envisage that studies employing isoform-specific antibodies or tagging isoforms endogenously using genome editing will be required to understand the intricate transcriptional biology of DAF-16 downstream of the IIS.

## MATERIALS AND METHODS

### Strain maintenance

Wild-type (N2 Bristol), *daf-2(e1370)* and *daf-16(mgDf50);daf-2(e1370)* mutant worm strains were obtained from Caenorhabditis Genetics Centre (Minneapolis, MN, USA). Throughout the manuscript, the *daf-16(mgDf50)* and *daf-2(e1370)* alleles are referred to as *daf-16(−)* and *daf-2(−)*, respectively. Worms were grown at 20°C unless otherwise mentioned.

### Generation of anti-DAF-16 antibody

The DAF-16 cDNA was amplified using primers CCCAAGCTTGGCCTATACGGGAGCAATGAGC and CCGCTCGAGCGGACGGAAAGATGATGGAACG and cloned in PET24b (EMD Millipore Biosciences, USA). The protein was expressed in BL21(DE3) strain of *E. coli* and purified using Ni-NTA column under denaturing conditions (8M urea) as per protocol provided by the manufacturer (Qiagen, USA). Step-wise dialysis was performed with the purified protein. After the final dialysis step in 1X PBS pH 7.5, much of the protein precipitated. The remaining protein that was left in the soluble form was used to immunize rabbits (250 μg per immunization, 6 boosters) to generate polyclonal antibodies.

### Chromatin immunoprecipitation (ChIP)

Mixed stage cultures of worms were grown on 15-20 *E. coli* OP50-seeded 150 × 15 mm NGM (Nematode Growth Media) agar petri-plates maintained at 20°C. A previously published ChIP protocol was used [7], with few modifications. Briefly, the worms were harvested from plates with 1 X PBS buffer and washed four times in the same buffer. The compact worm slurry (250 μl) was resuspended in 4 ml cross-linking buffer (1% formaldehyde in 1 X PBS) followed by homogenization with 7 ml glass Dounce homogenizer. Cross-linking was allowed to proceed for 15 min at room temperature. The homogenized worm lysate was quenched with 200 μl of 2.5 M glycine (125 mM final concentration) for 10 minutes. The worm pellet was washed four times with 1 X PBS, frozen in liquid nitrogen and stored at −80°C. The frozen pellet was resuspended and washed once in 2 ml of SDS lysis buffer (1% SDS, 10 mM EDTA and 50 mM Tris-Cl, pH 8.1) in presence of a protease inhibitor cocktail (Sigma, USA). The pellet was again resuspended in 2 ml of SDS lysis buffer and sonicated using Bioruptor Plus sonication device (Diagenode, Denville, NJ, USA) with output settings of 45 seconds on, 1 minute off at high intensity for 25 cycles in 10 ml tubes. The sonicated lysate was centrifuged at high speed for 20 minutes to collect the supernatant. Each lysate aliquot having 10 mg of protein was diluted 10 times with ChIP dilution buffer (1.1 % Triton X-100, 1.2 mM EDTA, 167 mM NaCl and 16.7 mM Tris-Cl, pH 8.0). One percent of the aliquot was saved as ‘Input sample’ and processed later along with ChIP samples. About 50 μl of salmon sperm DNA-coated protein A agarose beads (Millipore, USA) and 50 ul of pre-immune serum was added to the cell lysate and incubated for 1 hour at 4°C for preclearing. After centrifugation at 400 x g, 35 μl of anti-DAF-16 antibody was added to the precleared supernatant and incubated overnight at 4°C. Next day, the supernatant was incubated with 50 μl of salmon sperm DNA-coated protein A agarose beads for 2 hours at 4°C. The beads were transferred to a 1.5 ml micro-centrifuge tube, centrifuged at 400 x g and washed once with 1 ml of the low-salt-wash buffer (0.1% SDS, 1% Triton X-100, 2 mM EDTA, 150 mM NaCl and 20 mM Tris-HCl, pH 8.0), once with high salt wash buffer (same composition as of low salt wash buffer except 500 mM NaCl), once with LiCl was buffer (250 mM LiCl, 1% sodium deoxycholate, 1mM EDTA, 10 mM Tris-Hcl, pH 8.0) and three times with 1 X TE. ChIP-ed DNA was eluted with 500 μl elution buffer (1% SDS, 0.1 mM NaHCO_3_) by heating at 45°C for 10 minutes. Simultaneously, 400 μl of elution buffer was added to the input samples. 50 μl of 5M NaCl was added to each sample and kept for reverse cross-linking overnight at 65°C. Next day, 25 μl (1 mg ml^−1^) of DNase-free RNase (Roche Pharmaceuticals, Switzerland) was added and incubated at 37°C for 2 h. Then, 10 μl of 500 mM EDTA, 20 μl of 1 M Tris-HCl (pH-6.5) and 10 μl of proteinase K (20 mg ml^−1^) was added and incubated at 45°C for 2 h. The DNA was purified using phenol-chloroform and dissolved in 10 μl (for ChIP sample) or 30 μl (for input sample) of 10 mM Tris-Cl pH 8.0. The expected range of sonicated DNA was confirmed by running 3 μl of the input sample in a 2% agarose gel. The samples were stored at −80°C either for ChIP-PCRs or Next Generation Sequencing library preparations. All ChIP experiments were done with at least three biological replicates (each biological replicate with two technical replicates) and multiple samples were pooled for sequencing library preparation.

### Quantitative real-time PCR

The enrichment of DAF-16 on the promoter regions of known or newly identified genes was determined by quantitative real time PCR (qRT-PCR) using the Mesagreen MasterMix (Eurogentec, Belgium) and Realplex PCR system (Eppendorf, USA) according to manufacturer's specifications. The list of primers are provided in Table S6. The relative enrichment in *daf-2(−)* was determined after normalization with input and then compared with input-normalized *daf-16(−);daf-2(−)*. Fold change was calculated using ΔΔCT method [36] and statistical analysis was performed using SigmaPlot 10.0 (Systat software, USA).

### Construction of next generation sequencing libraries

ChIP-ed DNA (1 μl) was quantified with Quant-iT™ dsDNA HS Assay Kit in a Qubit^®^ fluorementer (Invitrogen, USA). A total of ∼10 ng of DNA was used as a starting material for library preparation according to the manufacturer's instructions (Illumina, San Diego, CA, USA). Briefly, the DNA was end-repaired, ‘A’ tailed and adapters were ligated to both ends. The DNA was purified using MinElute PCR Purification Kit (Qiagen, USA) and size-selected in the range of 200 ± 50 bp after running in a 2% UltraPure™ Low Melting Point Agarose (Invitrogen, USA). The DNA was extracted from the gel using a Gel Extraction Kit (Qiagen, USA), then PCR amplified for 18 cycles using Illumina-supplied PCR primers 1.1 and 2.1 and purified with Qiagen MinElute PCR Purification Kit. The library was validated using a High Sensitivity DNA Kit and High Sensitivity DNA Reagents on a 2100 Bioanalyzer (Agilent Technologies, USA).

### Next generation sequencing and analysis of ChIP-seq data

The ChIP or Input DNA was sequenced on a single lane of an Illumina Genome Analyzer IIx (GA IIx) for 36 cycles. Imaging, base calling and quality scoring were performed as per standard manufacturer's guidelines (Illumina, USA).

The de-multiplexing and conversion of BCL file format reads to FASTQ file format was done with Illumina-supported CASAVA v1.8.2 software package. Adapter and quality trimming was done with Cutadapt v1.2 using the parameters -m 15 -q 10 —quality-base 30. Quality-filtered reads were aligned to the *C. elegans* annotated reference genome (WS230) using Bowtie v.0.12.7 with parameters: -q -m 1 —best —strata. Peak calling was performed with uniquely-aligned reads, containing no more than one mismatch, using peak calling algorithm MACS v1.4.2 with parameters: —mfold = 5,30 —bw = 175 -w [37]. Statistically significant enriched peaks were selected with 5% FDR cut-off.

Assignment of DAF-16 peaks to the target genes was performed using PeakAnnotator, a PeakAnalyzer utility tool v1.4 [38]. The ChIP-seq peak summit (defined using MACS) was associated with a nearby gene transcription start site (TSS) and includes both coding as well as non-coding genes. In case of the presence of multiple genes in the vicinity of a summit, assignment was made to the closest TSS. For further analysis, peaks positioned within a window of 2.5 kb upstream or 300 bp downstream of a TSS were selected.

### Metadata analysis

Following peak calling using MACS, the location of the reads were shifted towards 3′ direction based on the mean fragment size [171 base pairs for WT, 158 base pairs for *daf-2(−)*, 200 base pairs for *daf-16(−);daf-2(−)*]. The genome was divided into 25-bp non-overlapping bins and the number of uniquely mapped reads in each bin was determined. The read counts per bin were normalized to the total number of uniquely mapped reads, both in the ChIP and input samples. The normalized input read counts were subtracted from the respective ChIP sample read counts, within each bin. To evaluate the average DAF-16 binding among different samples, the normalized genome-wide read counts were further quantile normalized, for statistical comparison, using preprocess Core R package. The bin containing each DAF-16 peak summit (determined by MACS) was identified and the mean normalized read count was determined for that bin. This procedure was repeated for 20 bins that were situated both upstream as well as downstream of the peak summit (25 bp × 20 = ± 500 bp). To show the spread of DAF-16 binding with respect to each peak, the above normalized read counts were plotted as heat map using MeV v.4.9. To visualize the aligned data as wig files (as in Figure 1A, S2), UCSC genome browser was used.

### *de novo* motif discovery

DAF-16 peaks assigned to promoters were used for *de novo* motif discovery. The sequences of the complete peak region were retrieved by Galaxy [39]. *De novo* motifs were identified using the peak motifs module of RSAT (Regulatory Sequence Analysis Tools) [18, 40] by using the following parameters: cut peak sequences ± 1000 bp, discover motifs with oligo and position analysis with oligomer length above 6-8 bp, Markov order (m = 2), five motifs per algorithm searched on both stands. We also analyzed the data using MEME (Multiple Em for Motif Elicitation) [41] and obtained similar results; only RSAT analysis is shown in the manuscript. Similar motifs were clustered with Cytoscape [42] by using the input files from RSAT. The motif with the highest number of edges and strongest correlation index was selected for further analysis. The selected motifs were then compared with the JASPAR [43] or TRANSFAC database Professional version 9.3 (http://www.biobase.de) to determine their identity.

### *In silico* validation of the discovered motifs

The discovered motifs were used to scan all the DAF-16 peak sequences (DAF-16 peak summits ± 250 bp) using the pattern matching module of RSAT, assuming that there is no more than one true binding site in the target sequence. Similar parameters were also considered for random promoters and genomic regions that were used as controls. Selections of the matched sequences were performed with a *P*-value ≤ 10^−5^ (based on the Markov model) with *C. elegans* reference genome WBcel230 as a background. The best match for the motifs (PSSMs match or weight score) was selected for downstream analysis including inter-motif distances *etc*. The statistical significance of the difference between the frequency of occurrence of the motifs within DAF-16 peaks lying in the promoter regions and the same number of randomly chosen promoter sequences was calculated using unpaired student *t*-test. We also calculated the ‘r’ or ratio of frequency of a motif's occurrence within the peak sequences to the random promoter sequences to evaluate the relative enrichment.

### Inter-motif distances

For this, the central co-ordinates of the sequences that matched the motifs were first determined. The relative position (distance) of the central co-ordinates of one motif was determined with respect to the other, with due consideration to the strandedness of the promoter. These distances were used to plot the histogram (Figure 6D, 6F) where DAF-16 motif positions were used as the reference point. Similar process was also followed to plot the relative positions of DAF-16 motifs with respect to peak summits (Figure S5A) and while comparing the relative positions of DAF-16 summits in *daf-2(−)* vs WT (Figure 3A).

### RNA-seq analysis and correlation with ChIP-seq data

For RNA isolation, mixed culture of worms was grown on *E. coli* OP_50_-seeded 90 mm NGM agar plates. The pellet was collected, after washing four times in M9 buffer, in 250 μl TRIzol reagent (Invitrogen, USA) and stored at −80°C. Total RNA was isolated as per manufacturer's recommendation.

Multiplexing was used while sequencing RNA with the help of indexed adapters as provided by manufacturer (Illumina, USA). Before library preparation, the quality of the RNA was checked on a 2100 Bioanalyzer by using the RNA 6000 Nano Kit (Agilent Technologies, USA). RNA with RNA integrity number (RIN) > 9 was selected for library preparation. The RNA library was prepared using the TrueSeq RNA SamplePrep V2 kit (Illumina, USA) according to manufacturer-provided specifications.

RNA-sequencing of WT, *daf-2(−)* and *daf-16(−);daf-2(−)*strains were performed using Illumina GAIIx. After de-multiplexing and adaptors trimming, reads were aligned to the annotated reference genome (WS230) using CLC Genomics Workbench v.6.5.1 and levels of the mapped genes (RPKM, Reads Per Kilobase of exon model per Million mapped reads) [44] were calculated using default parameters. Significant fold changes (*P*≤ 0.05, fold change ≥ 2) were selected by applying beta-binomial Baggerley's test [45]. Significance of overlap between different gene lists was calculated by hypergeometric distribution using GeneProf [46]. The fold enrichment of DAF-16-bound differentially expressed genes (DDEG) (*P*≤ 0.05, fold change ≥ 2) was calculated as follows: (number of DDEGs/number of DEG)/(number of DAF-16-bound expressed genes/number of expressed genes) (Figure 2B). Genes with RPKM >0 in the *daf-2(−)* and *daf-16(−);daf-2(−)* samples were considered as expressed genes. Further, the average mRNA fold change was calculated only for the genes having either single or double DAF-16 peaks within 0.5 kb promoter (Figure S1D).

### Data availability

The sequencing data is available to the readers at the following links:

ChIP-seq-GSE63865

http://www.ncbi.nlm.nih.gov/geo/query/acc.cgi?token=ktsdemoqjjuxpqb&acc=GSE63865

RNA-seq-GSE67975-

http://www.ncbi.nlm.nih.gov/geo/query/acc.cgi?token=ypwjukqithsljaz&acc=GSE67975

List of genes generated for detailed analysis shown in the manuscript is being provided as Data Set 1.

### Phenotypic analysis

#### Oxidative stress

Following hypochlorite treatment, eggs of *daf-2(e1370);rrf-3(pk1426)* were grown on RNAi-seeded NGM agar plates till they reached gravid adult stage. These worms were then transferred to respective RNAi plates that were overlaid with FUDR (final concentration of 50 μg/ml) and maintained at 20°C till Day 5 of adulthood. Approximately 10-12 adult worms were dispensed into each well of a 24-well tissue culture plate containing 400 μl of 100 mM paraquat (Sigma, USA) in 1X M9 buffer containing cholesterol. Worms were scored for survival at the 50th hour following the commencement of the experiment and those that failed to respond to gentle prodding were scored as dead. Data is presented as survival on 50th hour ± SD for each RNAi. Statistical analysis performed using Student's *t* test and is plotted in Figure 6C.

#### Heat stress

After hypochlorite treatment, eggs of *daf-2(e1370);rrf-3(pk1426)* were grown on the different RNAi as above. Approximately 100 animals per RNAi were upshifted to 30°C and scored for survival by gentle touching with a platinum wire every 6th hour. Statistical analyses for survival were conducted using Mantel-Cox log rank test through OASIS software available at http://sbi.postech.ac.kr/oasis [47] and plotted in Figure 6C.

#### Dauer

Dauer assay was performed at 22°C in the liquid culture containing RNAi feed. An RNAi mini-library was prepared for direct targets of DAF-16. A day before setting up the assay, the bacterial glycerol stocks from the library were inoculated in 500 μl LB containing 100 μg/ml ampicillin in a 96-deep-well plate and incubated for 16 hours at 37°C with shaking at 240 rpm. After incubation, the culture was induced for an hour with IPTG at a final concentration of 4mM. The culture was then pelleted and resuspended in 250 μl NGM containing 100 μg/ml ampicillin and 4 mM IPTG. About 60 μl NGM RNAi per well was dispensed into a flat-bottomed 96-well plate in triplicate. Approximately 10-15 L1 starved *daf-2*(*e1370);rrf-3(pk1426)* animals were added to each well in a maximum volume of 10 μl. The worms were then maintained at 22°C with constant shaking at 200 rpm. On 5^th^ day of the experiment, the worms were scored for dauer formation. The percentage dauer formation (6 wells for each RNAi) was calculated and compared to control RNAi-treated worms. Statistical analysis was performed using Student's *t* test and is plotted in Figure 6C.

#### Fat storage

Fat storage was determined in fixed worms using Oil Red O [48, 49]. Briefly, worms were synchronized using hypochlorite treatment and strains were grown on different RNAi plates till L4-YA stage. The worms were then washed and resuspended in 120 μl 1X PBS. To this an equal volume of 2X MRWB buffer (160 mM KCl, 40 mM NaCl, 14 mM Na_2_EGTA, PIPES pH 7.4, 1 mM Spermidine, 0.4 mM Spermine, 2% Paraformaldehyde, 0.2% beta-mercaptoethanol) was added and the same was incubated with shaking for 45 minutes. The worms were subjected to three freeze-thaw cycles in dry ice/ethanol bath, pelleted and washed with 1XPBS. Oil Red O was prepared as a stock solution 5mg/ml stock in isopropanol and equilibrated on a rocker shaker for several days. The working stock of Oil Red O was prepared by diluting the equilibrated stock to 60% using water and allowed to stand for 10 min following which it was filtered using a 0.22 μm filter. The Oil Red O stain was added to the fixed worms and the suspension incubated overnight on a shaker at room temperature. Following this, worms were washed twice with 1X PBS and mounted on 2% agarose slides for visualization using a AxioImager M2 microscope (Carl Zeiss, Germany) fitted with Axiocam MRm camera. The intensity of staining was quantified using NIH ImageJ software; statistical analysis was performed using Student's *t* test and plotted in Figure 6C.

## SUPPLEMENTARY FIGURES AND TABLES


















