# Supplementary material for: Genome-wide endogenous DAF-16/FOXO recruitment dynamics during lowered insulin signalling in C. elegans
Source: Oncotarget. 2015 Nov 2;6(39):41418–33. doi: 10.18632/oncotarget.6282 (PMC4747164; doi:10.18632/oncotarget.6282)
Supplement: Supplementary file 1 [file oncotarget-06-41418-s001.pdf]

# Genome-wide endogenous DAF-16/FOXO recruitment dynamics during lowered insulin signalling in *C. elegans*

## Supplementary Material

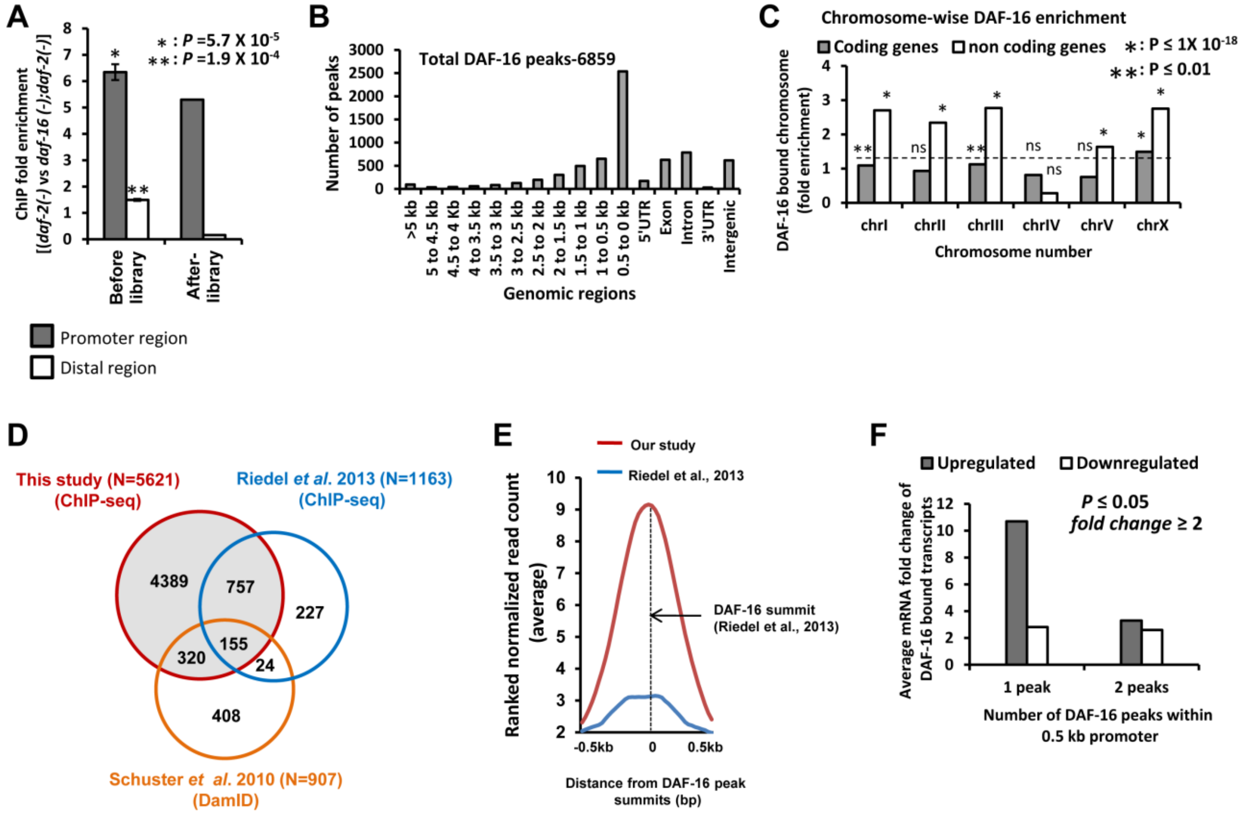


**Figure S1:** (A) ChIP was performed using *daf-2(-)* or *daf-16(-);daf-2(-)* cross-linked lysates. Quantitative real-time PCR (QRT-PCR) was used to determine the fold enrichment of DAF-16 binding to *sod-3* promoter (grey bar) or distal region (white bar) in *daf-2(-)* that was normalized to *daf-16(-);daf-2*, both before and after ChIP-seq library preparation. *P* calculated using Student's *t* test

(B) Distribution of all the DAF-16 peaks in *daf-2(-)*

(C) Higher enrichment of DAF-16 binding near non-coding genes on all chromosomes except chr-IV

(D) Comparison of genes that are bound by endogenous DAF-16 (this study) with earlier studies that employed either ChIP-seq [6] or DamID [5] using tagged DAF-16 overexpression strains

(E) Ranked normalized read counts in *daf-2(-)*from this study and that of previous ChIP-seq using overexpression strain [6] were plotted against the distance from the peak summits (± 0.5 kb).

(F) Average mRNA fold change of genes activated or repressed in *daf-2(-)*. The genes were categorized so that they either had one or two DAF-16 peaks within 0.5 kb proximal to TSS.

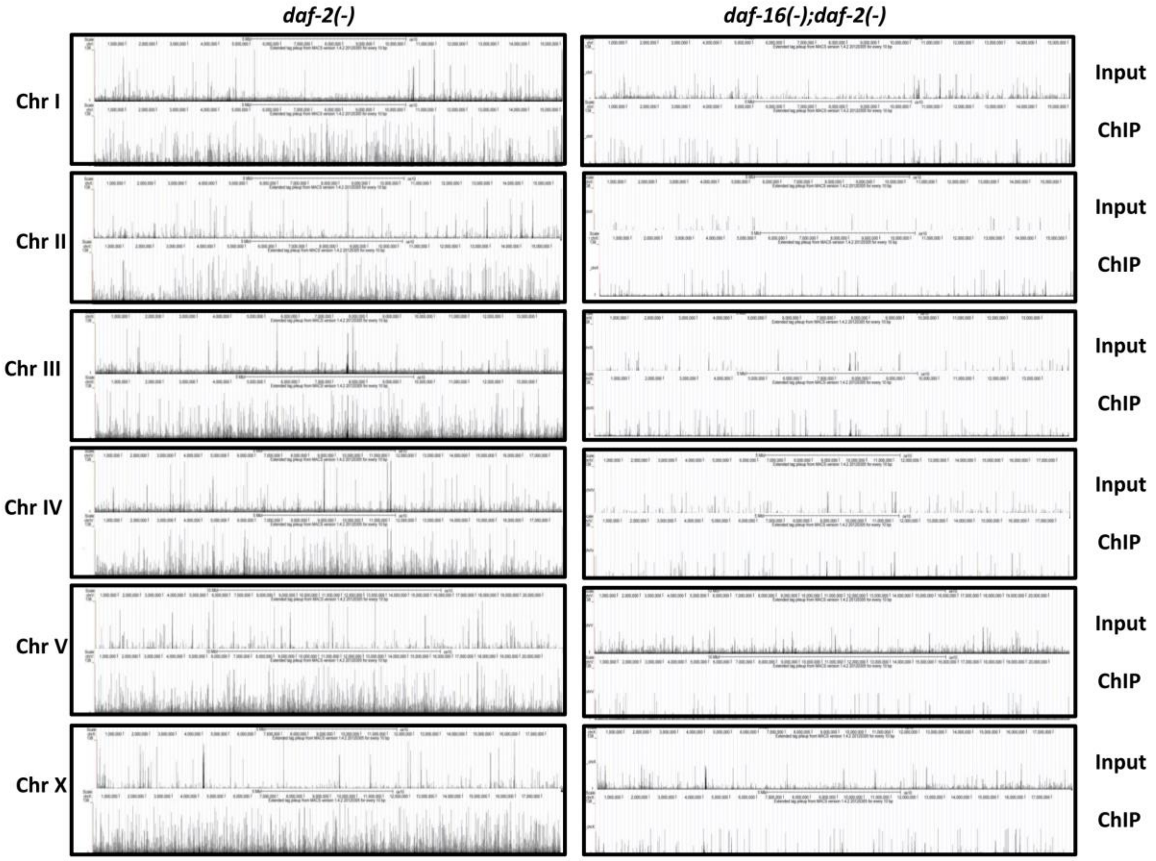


**Figure S2:** UCSC genome browser view of DAF-16 ChIP-seq data from *daf-2(-)* and *daf-16(-);daf-2(-)* for all the chromosomes showing enrichment of DAF-16 binding.

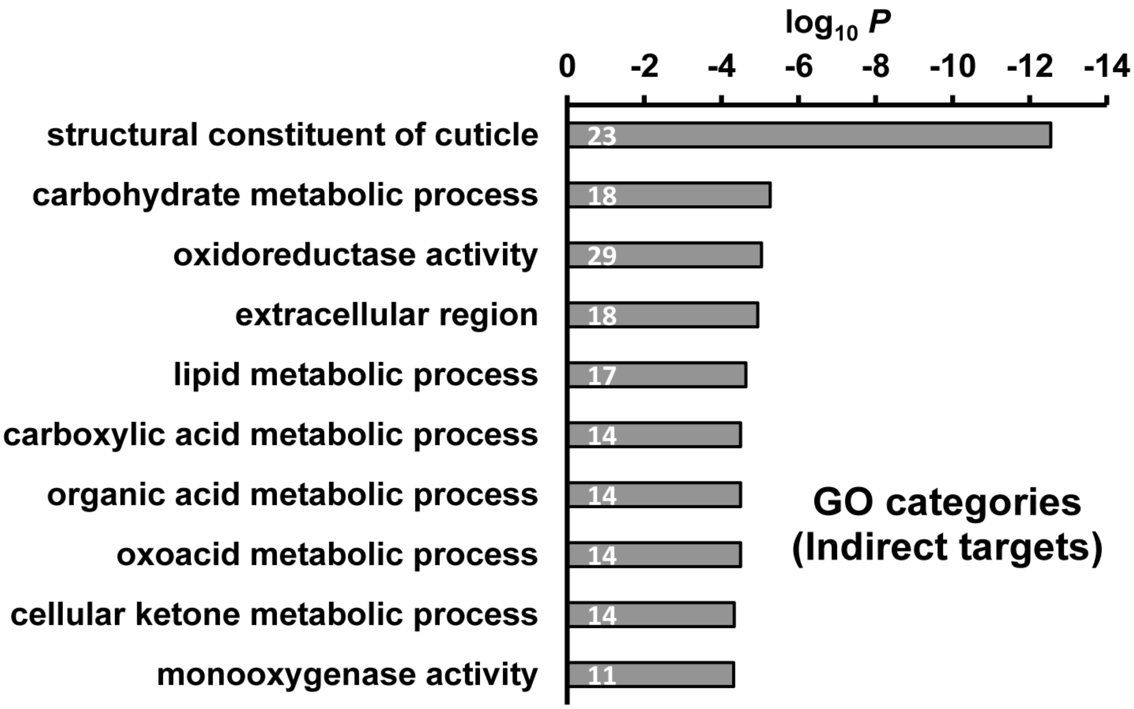


**Figure S3:** Gene Ontology analysis of DAF-16 indirect targets using DAVID. Genes functioning in metabolic processes are enriched.

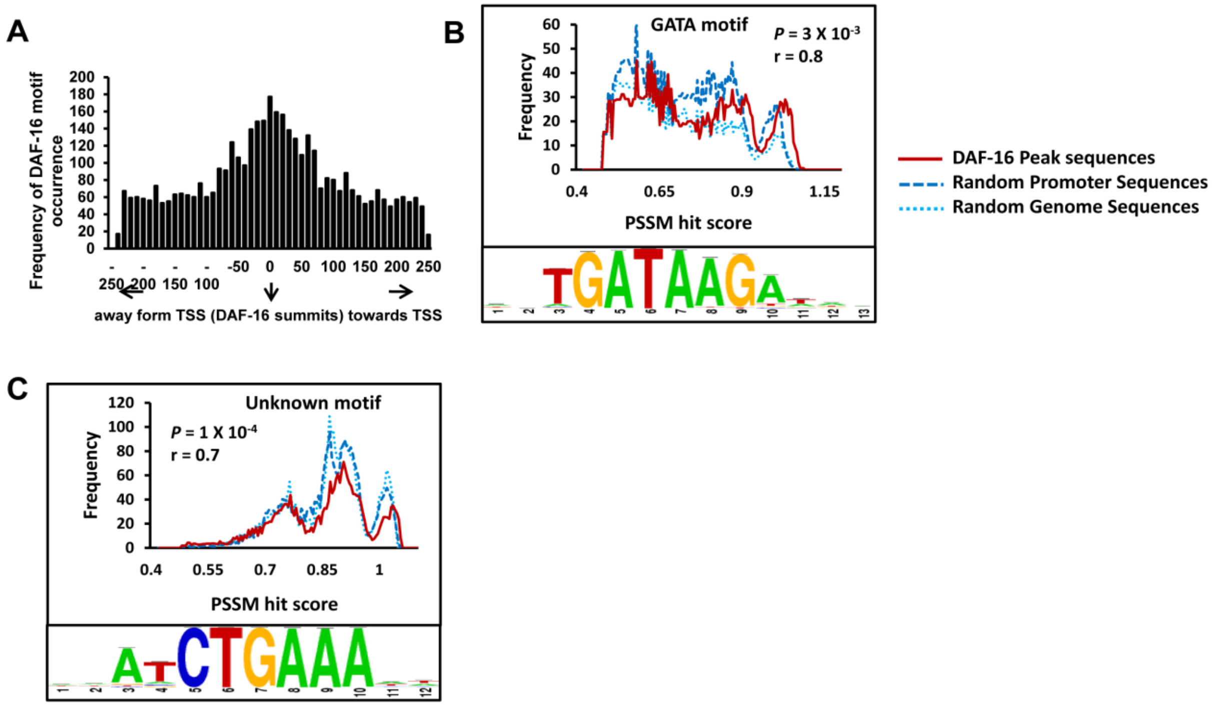


**Figure S4:** (A) Frequency of occurrence of DAF-16 motif with respect to DAF-16 summits. (B) Upper panel shows the frequency of GATA motif (red) within the DAF-16 peaks as compared to random sequences (blue). Lower panel contains the consensus GATA motif identified by RSAT.

(C) Upper panel shows the frequency of an unknown motif (red) within the DAF-16 peaks as compared to random sequences (blue). Lower panel contains the consensus of the unknown motif identified by RSAT.

*P* values calculated using unpaired student's *t* test.

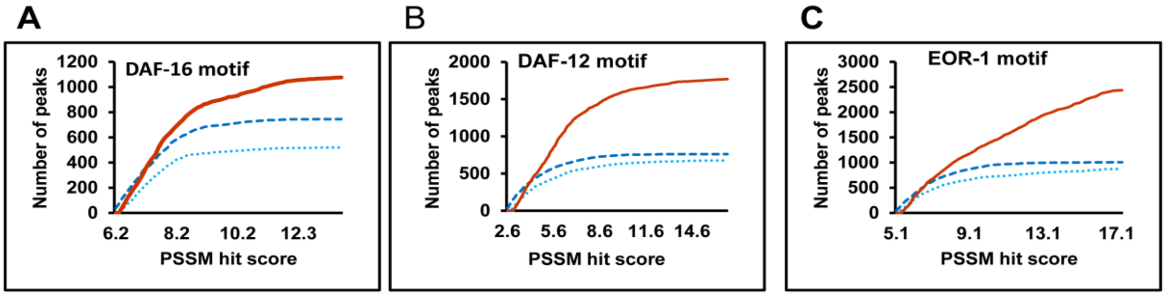


**Figure S5:** Graphs showing the number of DAF-16 peak having either DAF-16 (A), DAF-12 (B) or EOR-1 (C) motifs with respect to their PSSM hit score. The cut-off for PSSM hit score is $P \leq 1x10^{-4}$.

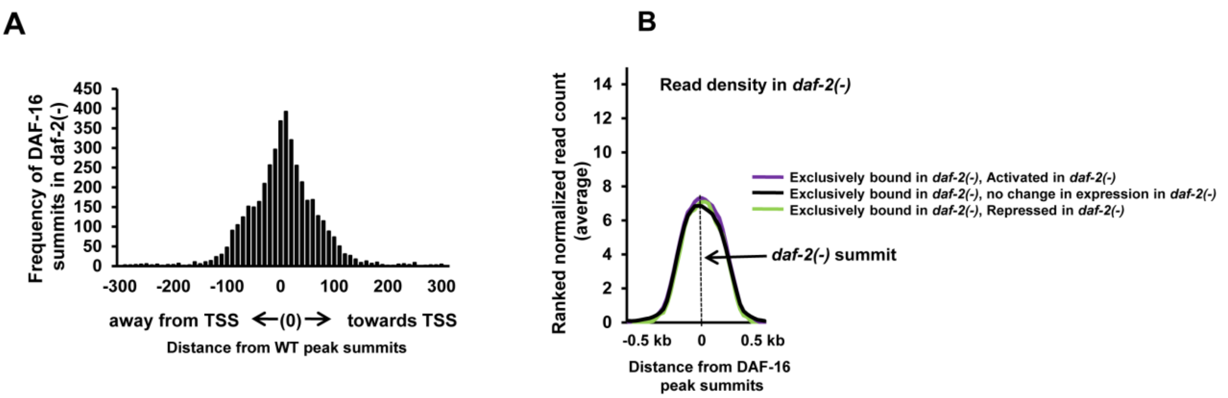


**C**

| Gene expression WT vs *daf-2(-)* | Representation factor (R): Binding vs expression of genes bound by DAF-16 in WT as well as in *daf-2(-)* | Representation factor (R): Binding vs expression of genes bound exclusively in *daf-2(-)* |
|---|---|---|
| Activated (N-667) | *2.2 (*P*=4.7 X $10^{-8}$) | *2.2 (*P*=3.2 X $10^{-23}$) |
| Random gene set (N-667) | 1.2 | 1.1 |
| Repressed (N-1213) | 0.8 | 0.6 |
| Random gene set (N-1213) | 1.1 | 1 |
| Unchanged (N-17518) | 1.0 | 1.0 |
| Random gene set (n-17518) | 1.0 | 1.0 |

**Figure S6:** (A) Histogram depicting the frequency of DAF-16 summits in *daf-2(-)* with respect to distance from the DAF-16 summits in WT (only peaks common in both WT and *daf-2(-)* were considered).

(B) Genes whose promoters are bound by DAF-16 exclusively in *daf-2(-)* have similar recruitment profiles irrespective of their expression levels compared to WT. Ranked normalized read counts in *daf-2(-)* were plotted against the distance from the peak summits (± 0.5 kb). These genes were categorised as activated, repressed or no change based on their expression in *daf-2(-)* compared to WT.

(C) Correlation of direct DAF-16 binding to the promoters of coding genes [bound exclusively in *daf-2(-)* or in both WT as well as in *daf-2(-)*] with their transcription. Numbers indicate representation factor (R). Genes that have DAF-16 bound to promoters under low IIS are significantly upregulated. R without *P* values are ≥ 0.01. * *P* values calculated using hypergeometric test.

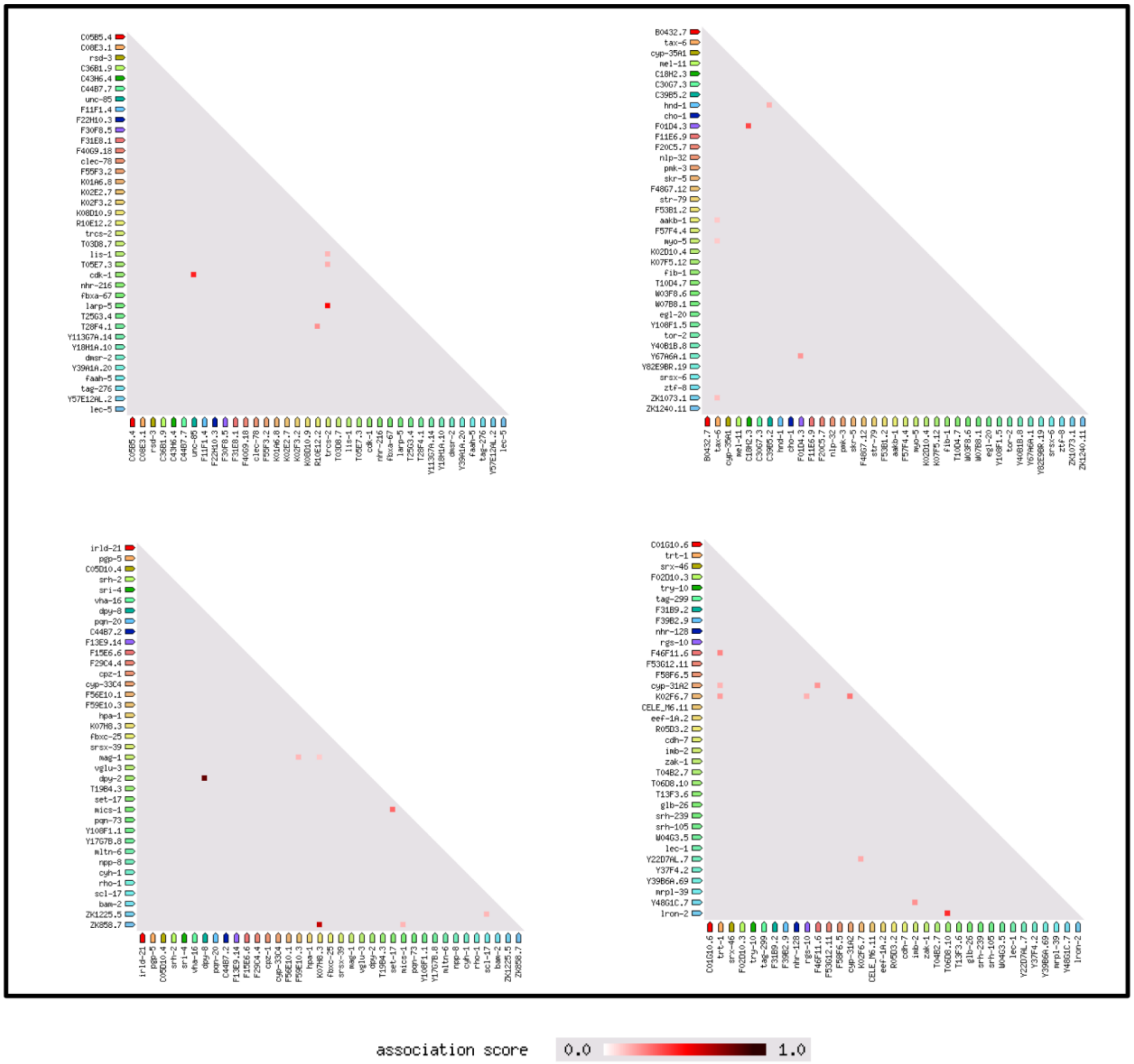


**Figure S7:** Co-expression analysis of four sets of randomly chosen 37 genes using STRING database version 10. No significant co-expression is noticed for most of the genes.

**List of Supplementary tables:**

Table S1: Summary of the ChIP-sequencing and mapping

Table S2: Summary of DAF-16 peaks

Table S3: Summary of RNA sequencing and mapping

Table S4: Second tier of regulators directly bound and activated by DAF-16

Table S5: Second tier of regulators directly bound and repressed by DAF-16

Table S6: Primers used in this study

Table S7: Phenotypic analysis following knocking down of 37 "core" DAF-16 direct targets. The P-values are plotted in Figure 6C.

Data set 1: All experimental details
